# Eagle Attack or Usual Transient Ischemic Attack? A Case Report

**DOI:** 10.51894/001c.165597

**Published:** 2026-07-31

**Authors:** Kamal Khalil

**Affiliations:** 1 DMC - Sinai Grace

## 81

### Background

Eagle syndrome (ES), particularly its vascular variant known as Stylocarotid artery syndrome (SAS), is characterized by an elongated styloid process (ESP) that impinges on the internal carotid artery (ICA), leading to vascular complications. The normal length of styloid process of temporal bone is 2 -3 cm. If larger, it can give rise to cervicofacial pain, dysphagia, dizziness or dysphonia. Historically, some patients have beneﬁted from NSAIDs, neuropathic pain agents, antidepressants, steroids, however surgery remains the mainstay of treatment. Here, we describe a case of recurrent TIAs over many years, likely secondary to styloid enlargement.

### Case Summary

An 80-year-old woman with past medical history of several TIAs, neck sprains, and hypertension presented with complaints of sudden aphasia and right hemiplegia which resolved completely enroute to the hospital, right cervicofacial pain persisted. She described a similar episode of sudden aphasia lasting 3 minutes about 2 days ago which resolved spontaneously. She also endorsed recurrent neck pain, occasional dysphonia and a sensation of fullness in the head, but no dysphagia, jaw pain, tinnitus or dizziness. CT head without contrast was negative for acute stroke or hemorrhage, however CT angiogram noted elongation of bilateral styloid processes: The right one measuring 7.1 cm and the left one 6.2 cm, raising suspicion for Eagle syndrome. Patient did end up having acute stroke during the course of hospitalization with small acute infarcts in left occipital and parietal lobes seen on MRI brain. Transesophageal echocardiogram was performed which did not reveal any cardioembolic source, right-to-left shunt, or valvopathy. So, 3D CTA of neck was undertaken and it showed elongated styloid process/calciﬁed stylohyoid ligaments abutting the external carotid arteries, & calciﬁcation with severe stenosis at the left external carotid artery origin. Considering numerous TIAs with complete resolution, recurrent cervical pain, CT evidence of elongated styloid processes, and no cardiac source of emboli on TEE, Eagle Syndrome continues to be a possible cause.

### Conclusion

Our case highlights need for guidelines regarding surgery for such patients with difficult-to-establish confirmation of Eagle Syndrome, but CT evidence of enlarged styloid processes (leading to increased thrombus formation) and clinical picture of multiple TIAs/Strokes.

